# Powerful linearly-polarized high-order random fiber laser pumped by broadband amplified spontaneous emission source

**DOI:** 10.1038/srep35213

**Published:** 2016-10-11

**Authors:** Jiangming Xu, Pu Zhou, Jinyong Leng, Jian Wu, Hanwei Zhang

**Affiliations:** 1 College of Optoelectronic Science and Engineering, National University of Defense Technology, Changsha 410073, China; 2 Hunan Provincial Collaborative Innovation Center of High Power Fiber Laser, Changsha 410073, PR China

## Abstract

A great deal of attention has been drawn to Random fiber lasers (RFLs) for their typical features of modeless, cavity-less and low coherence length. However, most previously reported high power RFLs employ narrowband fiber lasers as the pump source, thus inducing the self-pulsing transferring from pump source to output Stokes. In this contribution, linearly-polarized RFL pumped by broadband amplified-spontaneous-emission (ASE) is demonstrated and continuous-wave (CW) high order Stokes can be obtained.With 30.6 W pump injected into the half-opened cavity, 23.51 W the 2^nd^ order Stokes centered at 1178 nm with a full width at half-maximum linewidth of 1.73 nm and polarization extinction ratio of about 25 dB can be obtained. The standard deviation and peak-vale value of the 2^nd^ order Stokes light at maximal output power is just 0.47% and 4.10%, which indicates the good power stability. Significantly, the corresponding quantum efficiency of the 1^st^ and 2^nd^ order Stokes light is about 87% and 85%, and almost all pump photons are converted into Stokes photons. As far as we know, it is the highest power ever reported from linearly polarized RFL, and further power scaling is available in the case of more powerful pump source and optimization of system parameters.

Random lasers are of great importance in many fields, such as illumination source, sensing technology and spectroscopic monitoring for their typical features of modeless, cavity-less and low coherence length^1^,^2^,^3^. Random fiber laser (RFL) was first introduced in 2010^2^, which operate via extremely weak random scattering in a single mode fiber (SMF). The random distributed feedback (RDFB) is provided by backward random Rayleigh scattering and the amplification is given by the stimulated Raman scattering (SRS) effect. Recently, intensity investigations of RFL have been reported on the output characteristics of high power^4^,^5^,^6^, narrow linewidth^7^,^8^, spectral tunable^9^, pulsed operation^10^,^11^, and so on and so on^12^. Especially, linearly polarized laser is requested for some practical application fields such as sensing and communication.

The polarization properties of a RFL utilizing polarized pump was investigated for the first time in ref. 13, and modified power balance model was developed to qualitatively study the impact of polarization evolution on lasing power properties. Recently, single order and high order linearly polarized RFL pumped by polarization maintained (PM) fiber laser were presented with the maximum output power of about 10 W^14^,^15^. The PER of output Stokes reached as high as 25 dB, and the evolutions of power and spectral characteristics with the enhancement of pump power were studied in detail. In our previous work^16^, a linearly polarized random distributed feedback fiber laser was presented by introducing a certain fiber coiling technique. Despite of the depolarized pump source, the single linearly polarization laser output power reached ∼3 W with polarization extinction ratio (PER) >14 dB. But the PER value decreased to about 3 dB with the scaling of output power to 9.5 W for the lasing of the other polarization. As to the pump source, fiber laser is an extensive employed source for high power RFL^4^,^5^,^6^,^14^,^15^,^16^ for their high brightness, out-bound conversion efficiency, and maintenance free operation characteristics^17^,^18^. As we know, the demonstration of a truly continuous-wave (CW) fiber laser is, however, not trivial^19^. Usually, self-pulsing can occur in several kinds of fiber lasers, such as erbium-doped fiber laser^20^, ytterbium-doped fiber laser^21^,^22^, thulium-doped fiber laser^23^, Raman laser^24^, and even RFL^25^, under specific pumping and cavity conditions. When the fiber lasers with self-pulsing component are employed as the pump sources of RFL, the characteristic frequencies of self-pulsing in fiber lasers, usually in the range of kilohertz to hundreds of megahertz, can transfer to the output Stokes light of RFL^26^. By contrast, amplified spontaneous emission source (ASE, also referred to as superfluorescent fiber source) from rare-earth doped fiber has been proved to have high temporal stability^27^. And it is an ideal pump source for Raman laser^28^ and super-continuum generation^29^. Therefore, it’s significant to demonstrate more powerful linearly polarized RFL pumped by ASE light.

Here, we present a more powerful linearly polarized high order RFL pumped by high power ASE source and investigate the spectral-temporal evolutions in the power scaling process. Continuous-wave high order Stokes light can be obtained for the good stability of pump ASE source. Meanwhile, the dynamics of broadband emission spectrum and giant pulse operation at the thresholds of corresponding Stokes are described in spectral and temporal domain, respectively. 23.51 W the 2^nd^ order Stokes light centered at 1178 nm with a full width at half-maximum (FWHM) width of 1.73 nm and PER of about 25 dB can be obtained. To the best of our knowledge, this is the highest power linearly polarized RFL ever reported. Further power scaling is available in the case of more powerful pump source and optimization of system parameters.

## Results

**Figure 1:**
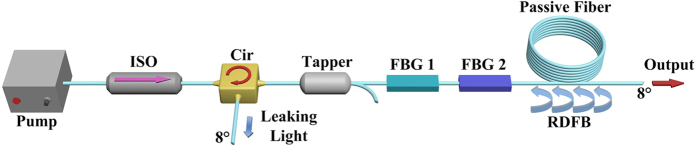
Experimental setup of the high power linearly-polarized random fiber laser. ISO: fiber isolator, Cir: circulator, FBG: fiber Bragg grating, RDFB: random distributed feedback.

The schematic diagram of the linearly-polarized RFL is depicted in Fig. 1. Linearly polarized broadband ASE light centered at 1073.5 nm with a FWHM linewidth of 12.5 nm is utilized to pump the linearly polarized RFL. To decrease the threshold of random lasing, half-opened cavity structure^9^,^30^ is employed, which is composed by two narrowband fiber Bragg gratings (FBGs) and a piece of 500 m long PM passive fiber. The FBGs are utilized to select the radiation section of initial spontaneous Raman noise to obtain narrowband output. The passive fiber is employed to provide random distributed feedback and Raman gain for the random lasing, and the attenuation ratio for the broadband pump light is about 13%.

**Figure 2:**
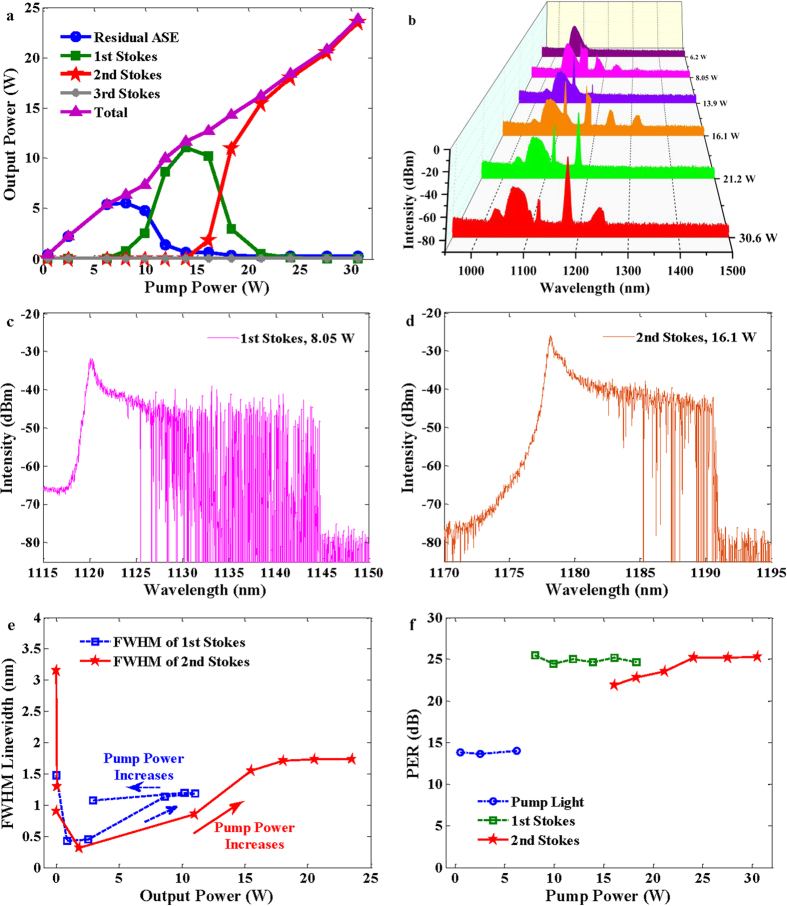
Output characteristics of the ASE pumped linearly polarized RFL. (**a**) Output power as a function of injected pump power; (**b**) Output spectra at different pump power; (**c,d**) Spectral details of the 1^st^ and 2^nd^ Stokes at corresponding threshold, (**e**) FWHM linewidth of Stokes waves as a function of its output power; (**f**) PER of the transmitted light as a function of injected pump power. The output spectra are measured with a resolution of 0.02 nm.

The output power and spectrum characteristics of the RFL at different pump power levels have been measured and depicted in Fig. 2(a,b), respectively. Below the lasing threshold of the 1^st^ stokes (8.05 W), only transmitted pump can be observed in the output light. With the enhancement of pump power from 8.05 W, the 1^st^ Stokes power grows rapidly with the pump power up to the 2^nd^ Stokes threshold (16.1 W) and then starts to decrease. As shown in Fig. 2(b), broad emission spectra of 1^st^ and 2^nd^ stokes wave can be observed, and several high order Stokes are excited near the threshold of the 1^st^ Stokes and 2^nd^ Stokes. The details of the broadband 1^st^ and 2^nd^ Stokes generated at corresponding thresholds are displayed in Fig. 2(c,d), respectively. The FWHM linewidth of the Stokes light near corresponding threshold is as narrow as 0.42 nm and 0.31 nm, respectively. But the emission spectrum of Stokes light covers the range of 1118~1145 nm for the 1^st^ Stokes light and 1170~1190 nm for the 2^nd^ Stokes light, which is typical for the multi-cascaded Rayleigh scattering (RS) - stimulated Brillouin scattering (SBS) generation^2^,^31^. The broadband shapes of the 1^st^ and 2^nd^ Stokes waves will evolve to narrowband shape when pump power exceeds threshold level. The temporal dynamics of these evolutions will be investigated in detail later. Additionally, the compositions around 1030 nm, as shown in Fig. 2(b), are induced by the power amplification process in the pump source. The ultimate total output power of the RFL is 23.8 W for given 30.6 W pump light, which is limited by the available pump power. At full pump power, almost all pump light converses to Stokes light, and the residual ASE is just about 0.28 W. Subtracting the residual pump power, the pump-to-Stokes conversion efficiency is about 77.57%. The individual highest pump-to-Stokes conversion efficiency during the power enhancing process for the 1^st^ and 2^nd^ Stokes is 83.43% and 77.55%, respectively. And the corresponding quantum efficiency is about 87% and 85%, respectively. To the best of our knowledge, this is the highest power linearly polarized RFL ever reported, and further power scaling is available in the case of more powerful pump source and optimization of system parameters.

The spectral linewidths of the 1^st^ and 2^nd^ Stokes as a function of its output power are shown in Fig. 2(e). Below the threshold of the 1^st^ Stokes, the FWHM linewidth of the 1^st^ Stokes is about 1.48 nm, which is approximate to the FWHM linewidth of FBG 1. The FWHM linewidth of the 1^st^ Stokes decreases to about 0.42 nm around threshold level, and broadens to a stable value of about 1.2 nm with the increment of pump power. As to the 2^nd^ Stokes, the initial FWHM linewidth is about 3.15 nm, and it decreases to 0.31 nm around threshold. With the enhancement of output power, the FWHM linewidth evolves to a stable value of about 1.73 nm. Below the threshold, initial output spectrum of individual Stokes is formatted by the interaction between the reflection of FBG and spontaneous Raman emission, and the FWHM linewidth of output spectrum is determined by the linewidth of the narrowband FBG. With the increment of pump power to individual lasing threshold of 1^st^ and 2^nd^ Stokes, Schawlow-Townes spectral narrowing^32^ can be observed as global statistically stationary wave spectrum formatted. Above the individual lasing threshold, nonlinear broadening will occur for the effects such as self-phase modulation (SPM) and cross-phase modulation (XPM)^15^. Figure 2(f) plots the evolution of PER of the transmitted pump light and the 1^st^ and 2^nd^ Stokes light. The PER of the random lasing output can achieve as high as about 25 dB even at maximal pump power, despite of the low PER value of pump light (<14 dB). It indicates that the random lasing process in the linearly polarized RFL can improve the polarization characteristic for the difference of Raman gain on the two polarization axis.

**Figure 3:**
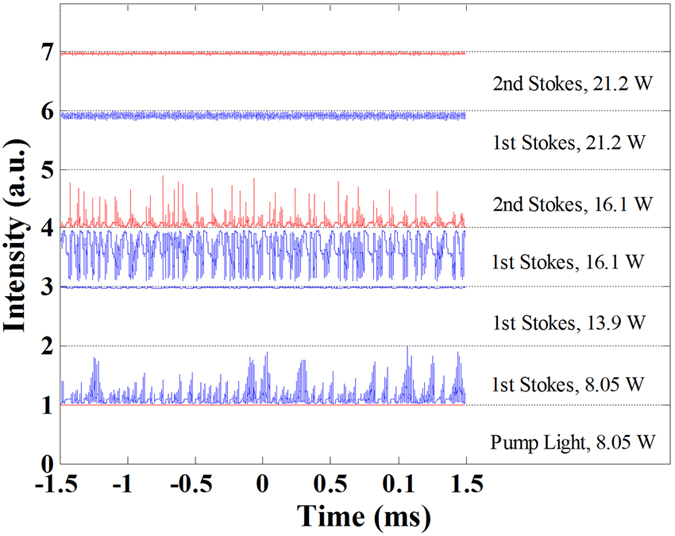
Evolution of ASE pumped RFL at different pump power. The temporal data of individual components are measured and acquired synchronistical with a time resolution of 5 ns.

The temporal characteristics of the linearly polarized RFL pumped by broadband ASE at different pump power are depicted in Fig. 3. To compare the power fluctuation amplitude and intensity of the output light, standard deviation (STD) and peak-vale (PV) values of the temporal measurements are calculated. For the good stability of broadband ASE light^27^, the STD and PV value of the pump ASE at 8.05 W is 0.18% and 1.58%, respectively. The good power stability maintains well in the power scaling process of pump light. At the corresponding threshold of the 1^st^ and 2^nd^ order Stokes light (8.05 W and 16.1 W, respectively), giant pulse operation generates and vanishes with the increment of pump power. The dynamics of this temporal evolution have been intensive investigated^33^,^34^,^35^. With the scaling of pump power from 8.05 W to 13.9 W, stable Stokes light can be obtained. The STD and P-V values are 0.35% and 3.36%, respectively. At the threshold of the 2^nd^ Stokes (16.1 W), the output of 1^st^ and 2^nd^ Stokes light start to operating unstable. The evolutions of the 1^st^ and 2^nd^ Stokes are synchronized and the tendencies are opposite, indicating that the cause of pulse operation of 1^st^ Stokes light is the energy consumption of pulse radiation of the 2^nd^ Stokes. The giant pulse lasing of linearly polarized RFL at the threshold of 2^nd^ Stokes can transform to stable operation again with the increment of pump power to 21.2 W. The STD of the 1^st^ and 2^nd^ Stokes light is 2.15% and 0.82%, and the P-V value is 20.34% and 7.27%, respectively. The power stability of the 2^nd^ Stokes light is better than the 1^st^ Stokes light, but worse than the broadband pump ASE. At full pump power, the STD and P-V values of the output 2^nd^ Stokes light is 0.47% and 4.10%, respectively. In contrast to the good stability of pump light, the causes of the power stability deterioration maybe lie in Raman noise and influence from the environment.

## Discussion

For the good stability of broadband ASE light, CW operation of high order linearly polarized Stokes light with pump-limited maximal output power of 23.8 W and FWHM linewidth of about 1.73 nm can be obtained from linearly polarized RFL pumped by broadband ASE source. However, giant pulse operation and broadband spectrum radiation can be observed at corresponding threshold, which is different to the fiber laser pumped linearly polarized RFL^14^,^15^. The occurrence of giant pulse in high power linearly polarized RFL cannot be ignored for the potential irreversible damages on the fiber, such as fiber fuse. Further investigation on the characteristics of giant pulse operation and the suppression method, even eliminate the giant pulse generation is significant for the demonstration and optimization of high power CW operated RFL. What’s more, the corresponding threshold of the 1^st^ and 2^nd^ Stokes generation is about 8.05 W and 16.1 W, which is almost two times higher than that of the presented linearly polarized RFL^14^ with similar operation parameters and pumped by narrowband fiber laser. The threshold enhancement maybe lies on the differences of temporal characteristics and FWHM linewidth of pump source. As to the efficiency characteristics, the individual optical-to-optical conversion efficiency of pump to 1^st^ and 2^nd^ order Stokes light is 83.43% and 77.55%, respectively. The corresponding quantum efficiency is about 87% and 85%, accordingly. Considering the attenuation loss induced by the passive fiber, almost all pump photons are converted into the Stokes photons. The obtained quantum efficiency for the 1^st^ order Stokes light (87%) is lower than that of reported non-polarized RFL (about 93%)^4^ and presented linearly polarized RFL (about 92%)^14^. The relative low quantum efficiency of this scheme mainly lies on the high cavity loss while the cavity loss is only about 4% and 10% for the RFL in refs 4 and 14, accordingly. Furthermore, the quantum efficiency of converting the pump light to the 2^nd^ order Stokes light in this system sets record value (85%) in high order RFLs comparing with the quantum efficiency in previous non-polarized high-order high efficiency RFLs (59%)^36^ and linearly polarized high order RFL with ultimate efficiency (83%)^15^. The relative low quantum efficiencies of the reported high order RFLs are influenced by the relative high cavity loss. And the corresponding cavity loss of the high order RFL demonstrated in refs 15 and 36 is about 46% and 15%, respectively. The quantum efficiency obtained in this scheme indicates that high conversion efficiency can also be realized in high order linearly polarized narrowband RFL pumping by broadband ASE source (12.5 nm FWHM linewidth). The FWHM linewidth of the 2^nd^ order Stokes light is 1.73 nm and slightly narrower than the FWHM linewidth of FBG2, which may be induced by the spectrum mismatch of FBG2 and spontaneous Raman light. What’s more, the FWHM linewidth of the 2^nd^ order Stokes light is also narrower than that of reported results (about 2.5 nm at maximal output power)^15^. Firstly, the narrowband FBGs acting as spectral filters can reduce the linewidth of output Stokes light directly. Secondly, the difference of the linewidth maybe also owed to the shorter passive fiber employing in this scheme as spectrum broadening is induced by nonlinear effects such as SPM and XPM^15^, and the fiber length can influence the generation of nonlinear effects.

## Conclusion

In this contribution, we demonstrated a linearly polarized high order RFL pumped by broadband ASE source. Half-opened cavity, which is composed by two high reflectivity FBGs and a piece of 500 m long single mode PM passive fiber, is employed to decrease the threshold of Stokes light generation. The threshold of the 1^st^ and 2^nd^ order Stokes light is 8.05 W and 16.1 W, respectively. At corresponding threshold, giant pulse operation and broadband spectrum emission generates as SBS-RS combined effect. The dynamics of these phenomena are explained in spectral and temporal domain, respectively. With 30.6 W pump light injected, maximal output power of 23.51 W CW second-order Stokes light centered at 1178 nm with a FWHM of 1.73 nm. The quantum efficiency of pump to the 1^st^ and 2^nd^ order Stokes light is about 87% and 85%, respectively. Considering the attenuation loss induced by the passive fiber, almost all pump photons are converted into the Stokes photons. The PER of the Stokes light can achieve as high as about 25 dB even at maximal pump power, despite of the low PER value of pump light (<14 dB). The STD and P-V value of the output 2^nd^ Stokes light at maximal output power is just 0.47% and 4.10%, which indicates the good power stability of output high order Stokes light. To the best of our knowledge, this is the highest power linearly polarized RFL ever reported. Further power scaling is available in the case of more powerful pump source and optimization of system parameters.

## Methods

The experimental setup is schematically depicted in Fig. 1. Pump sources we employed are homemade main-oscillator-power-amplifier (MOPA) structured linearly-polarized fiber sources seeded with broadband CW operated ASE source. To increase the isolation between the pump source and the random laser and avoid the influence of the leaking light of the FBGs, PM fiber isolator (ISO) and circulator are cascaded fused after the pump source, and the backward light of the random fiber laser is striped from the 3^rd^ port of fiber circulator. The end facet of the 3^rd^ port is angle cleaved with 8° to decrease the Fresnel reflection. A PM tapper is utilized to monitor the temporal characteristics of pump light injected into the RFL. The coupling ratios of the PM tapper to monitor port and signal port are 0.1% and 99.9%, respectively. The half-opened cavity of the linearly polarized high order RFL is composed by a piece of 500 m long single mode PM passive fiber and two PM high reflectivity FBGs centered at 1120 nm and 1178 nm, respectively. The core diameter of the passive fiber is 6 μm, and the corresponding FWHM linewidth of FBG 1 and 2 are 1.41 nm and 2.16 nm. The end facet of the output port of passive fiber is angle cleaved with 8 °C to suppress the point reflection.

The output power and spectrum are measured by a power meter and an optical spectrum analyzer (OSA). The PER of the transmitted light is measured by spatial PER measurement. After the collimation of output beam by a spatial collimator, high reflection mirror operated at 1070 nm with a bandwidth of ±10 nm and dichroic mirror for 1120 nm/1178 nm wavelength are utilized to pick out the residual pump light from the Stokes light and split the high order Stokes light. Half-wave plates and polarization beam splitters operating at corresponding wavelength are employed after the spectrum section splitting. The power along the two polarization axis are measured by two power meters and the PER value can be calculated. The temporal properties of the individual Stokes light and pump source are measured by InGaAs photodetectors (5 GHz bandwidth, rise time <70 ps) and digital phosphor oscilloscope (1 GHz bandwidth, 5 GS/sec sampling rate) with the help of spectrum splitting mirrors and fiber tapper.

## Additional Information

**How to cite this article**: Xu, J. *et al.* Powerful linearly-polarized high-order random fiber laser pumped by broadband amplified spontaneous emission source. *Sci. Rep.* **6**, 35213; doi: 10.1038/srep35213 (2016).
